# Food for thought or food for emotions? An analysis of marketing strategies in television food advertising seen by children in Colombia

**DOI:** 10.1017/S1368980023001702

**Published:** 2023-11

**Authors:** Alcides Velasquez, Maria Fernanda Parra, Mercedes Mora-Plazas, Luis Fernando Gómez, Lindsey Smith Taillie, Francesca Renee Dillman Carpentier

**Affiliations:** 1 Communication Studies Department, University of Kansas, Lawrence, KS, USA; 2 Facultad de Medicina, Pontificia Universidad Javeriana, Bogotá, Colombia; 3 Departamento de Nutrición Humana, Universidad Nacional de Colombia, Bogotá, Colombia; 4 Carolina Population Center and Department of Nutrition, Gillings School of Global Public Health, University of North Carolina at Chapel Hill, Chapel Hill, NC, USA; 5 Hussman School of Journalism and Media, University of North Carolina at Chapel Hill, Chapel Hill, NC 27599, USA

**Keywords:** Food marketing, Food advertising, Childhood obesity, Nutrition transition, Commercial determinants of health

## Abstract

**Objective:**

To establish the relationship between the marketing strategies and nutritional quality of foods and beverages promoted in television food advertisements (TV ads) seen by Colombian child audiences overall and based on child gender and socio-economic strata (SES).

**Design:**

A quantitative content analysis of marketing appeals was combined with nutritional data of the food products advertised and matched with TV audience ratings data for each food and beverage ads for Colombian children between 4 and 11 years.

**Setting:**

All beverages and foods TV ads cable or over-the-air channels in Colombia in 2017.

**Participants:**

N/A.

**Results:**

Compared with rational appeals (e.g. freshness, health or nutrition messages), emotional appeals (referencing or depicting human senses or emotions, e.g. using cartoons to suggest fun) were more frequently used in the TV ads most viewed by Colombian children. Female children and children in lower SES tended to be more exposed to emotional appeals in TV ads than their male or higher SES counterparts. Furthermore, TV ads using more emotional appeals tended to be for products high in problematic nutrients.

**Conclusion:**

The findings of this study highlight the need to implement statutory measures to reduce the deleterious effect of food marketing on children.

In Colombia, as in other Latin American countries, the prevalence of obesity among children has continued to grow^(1,2)^, due in part to social, cultural and economic transformations in the region over the last decades^(3,4)^. There is compelling evidence that obesity increases the risk of depression, anxiety, low self-esteem, bullying victimisation, eating disorders and poor school performance^(5–7)^. Moreover, studies show that children with obesity are more likely to remain obese and develop type 2 diabetes and CVD as adults^(8,9)^.

Multiple macro and micro-level factors induce children’s obesity and overweight^(10)^. According to several systematic reviews, food marketing is a major commercial determinant of childhood obesity, closely interwoven with other key industrial practices such as political interference and reputational management^(11–14)^. The extent of exposure to food advertising and the persuasive power of food ads have both been found to influence children’s consumption of high caloric and low nutritional value food products – which importantly contribute to childhood obesity^(15,16)^. Exposure refers to the number of people reached and the frequency with which these people are exposed to marketing messages, and persuasive power pertains to the content and design characteristics of the message^(17)^. This study aims to investigate how Colombian children are influenced by television advertisements (TV ads) promoting foods and beverages of differing nutritional quality, in terms of their exposure to and the persuasive power of these ads.

In terms of the advertising power, this study analyses two persuasive appeal strategies in food and beverage TV ads: *emotional* and *rational* appeals. Generally, persuasive appeals influence consumers by generating a positive association with the product and with the consumption experience^(18)^. *Emotional appeals* influence children by creating associations between the product and emotions like fun, happiness, excitement, sense of imagination or adventure^(19)^. Emotional appeals directed towards children are known to convey positive emotions with cartoon and licensed characters, child actors and other animations that attract children’s attention^(20)^ and, more recently, social media influencers^(21)^. Emotional appeals have been observed to be frequently used in child-directed TV food ads, likely because of evidence that they generate favourable impressions of the advertised product^(19,22)^. Children are susceptible to emotional appeals due to their developmental stage marked by less careful information processing and an emphasis on instant gratification and reward^(23)^.

In contrast, information about the attributes of a food product or claims about the product’s nutritional advantages, called *rational appeals*, is thought to be less attractive to children and more likely used as adult-directed persuasive strategies^(24)^. Rational appeals rely on the expectation that consumers will process information about a product through a logical and rational process^(19,25)^. Thus, rational appeals focus on arguments about the advantages and benefits of a particular product and not on the emotional experience of consuming the product.

The present study aims to document differences in how emotional and rational appeals are used in the TV food ads to which children in Colombia are most exposed. By doing so, this study provides two contributions to the literature. First, this study describes a methodology to examine in detail the use of different appeal types classified as emotional and rational appeal strategies. Existing methodologies for monitoring and studying the advertising power of food and beverage TV ads include a limited number of appeal types for each of these strategies^(26)^. Therefore, this study describes a codebook designed specifically to differentiate between emotional and rational appeal types based on prior research.

Second, this study provides empirical evidence of the marketing power of food and beverages in the TV ads to which Colombian children are heavily exposed, based on the nutritional quality of those advertised products. This information is critical to understanding how children are being influenced by food marketing in TV. In Colombia, TV remains the most widely used communication medium with an estimated 91·4 % of households owning at least one TV set, compared with 39·3 % owning a desktop, laptop or tablet computer. Children on average spend between 140 and 200 min watching TV on weekdays and weekends, respectively^(27)^. As such, TV advertising is an effective way to reach young consumers. However, studies have shown that TV food advertising to which Colombian children are exposed promotes processed and ultra-processed food and beverages over non-processed food products^(27,28)^. Thus, Colombian children are exposed to more TV commercials for products of low nutritional quality than more nutritious products^(29)^. Additionally, TV advertising can impact children’s dietary patterns and contribute to the increasing prevalence of overweight and obesity in Colombian children.

Furthermore, higher obesity levels have been observed among Colombian children from higher socio-economic strata (SES) than those from lower SES^(30,31)^, with boys experiencing more overweight and obesity than girls^(31)^. On the other hand, more adult women and individuals from lower SES have obesity compared with men and those from higher SES^(31)^. These patterns highlight the importance of examining potential disproportionate exposure to different marketing strategies, this is rational and emotional appeals, by male and female children and those from different SES, given the link between childhood obesity and obesity in adulthood.

To address these issues, this study addresses the following four research questions:

1. What are the most frequently used persuasive appeal strategies promoting food and beverage products among the TV ads to which Colombian children are more exposed?
2. Is there a relationship between the nutritional properties of food and beverage products and the persuasive appeals used to promote them among the TV ads to which Colombian children are more exposed?
3. Is there a difference between Colombian female and male children in their exposure to different persuasive appeal strategies in TV food and beverage ads?
4. Is there a difference between Colombian children from low, middle and high (SES) in their exposure to different persuasive appeal strategies in TV food and beverage ads?

## Method

### Procedure for determining television advertisements with the highest exposure

A quantitative content analysis was performed on the top 20 % of the TV ads with the highest levels of exposure in 2017 for Colombian children between 4 and 11 years. The methodology used to sample and examine these TV ads was derived from adaptations from existing protocols for monitoring food advertising^(26)^ and content analyses specific to the Latin American^(20)^ and Colombian^(1,29)^ contexts. A data set with the list of all TV ads was acquired from the media research company Kantar IBOPE Media. The data set included information about child audience sizes for each ad via rating point estimates of each instance of each TV ad shown on Colombian TV during 2017. One rating point for a given piece of content means that 1 % of the total target audience viewed that piece of content. In addition to rating points for the overall child population, this initial data set also included rating points information for male and female children and for children living in low, middle and high SES. Further information included the date and time of each TV ad instance, the duration of each ad, the advertised product, product brand, the TV channel in which the ad was featured and the TV show in which the ad was placed.

The sample of top ads was derived from this initial data set of all ads placed on all cable and over-the-air TV channels in Colombia between 06.00 and 22.00 hour throughout the year. This approach ensured that the sample of those commercials with the highest level of exposure during the entire year 2017 was determined independently of the TV programme, channel, time of day, day of the week and time of year, allowing a level of specificity and granularity not obtained with methods that focus on only certain channels or programmes^(32)^.

**Fig. 1:**
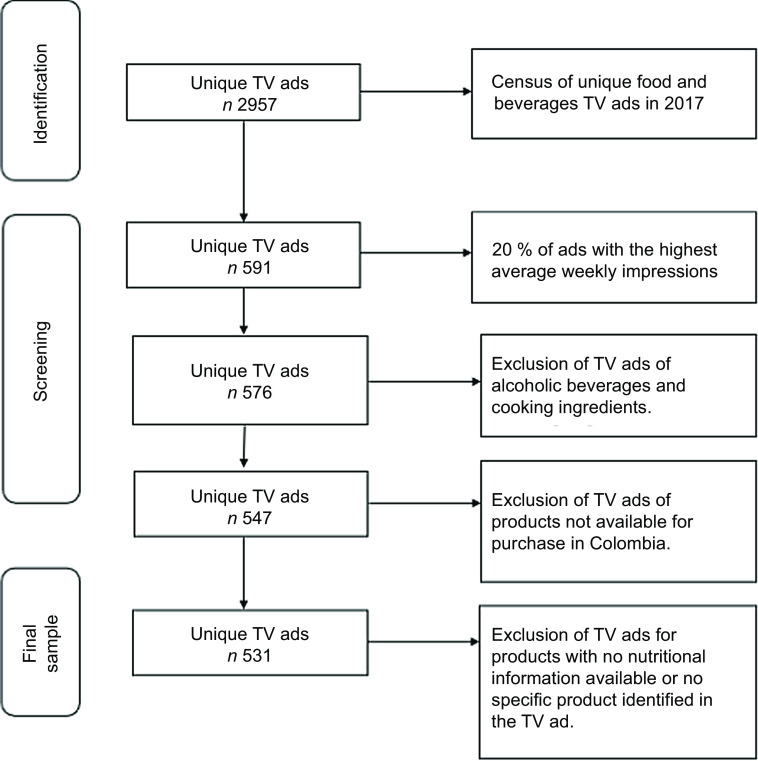
Sample construction flow chart

Rating points were used to identify ads with the highest levels of child audience exposure, defined as the number of individuals reached and the frequency of reach throughout the entirety of the year. That is, ads were selected for analysis based on records of exposure for children between 4 and 11 years old and not on estimates of future exposure as used in some other methodologies^(26)^. Figure 1 includes a flow chart illustrating the process for sampling television ads for the content analysis.

Although not infallible, the possession of data on the date, time and duration of each TV ad instance for the whole year allowed for the verification of the completeness of the list of commercials provided by Kantar IBOPE Media. Similarly, by establishing our sample based on TV ads with the highest exposure throughout the year, the possibility of excluding specific instances of advertisements and the impact of their potential exclusion on the constitution of the sample was minimised.

The first step in the building of the sample was to determine the *unique* TV food and beverage ads to which children were most exposed during 2017. First, all instances of the same TV ad were grouped together, including TV ads with multiple versions that differed in their duration. This resulted in a total of 2957 unique TV ads and 603 716 TV ad instances (total number of ads, including repeat ads) for the year.

Noted above, Kantar IBOPE Media provided the child 4–11 years old audience rating points for each instance of each ad (1 rating point = 1 % of total child TV audience). Therefore, the next step was to calculate the average rating points per unique TV ad. This was done by summing the rating points from each instance of the unique ad and dividing the sum by the number of instances for that ad.

In order to calculate an estimate of exposure for each unique TV ad, we adopted a metric that incorporated both the number of children reached and the frequency with which they viewed the content, the two components of the definition of exposure adopted in the study. To do this, we calculated the average weekly impressions for each unique TV ad. Impressions are one of the metrics used in advertising to calculate how much a specific audience is exposed to media content, defined as the number of times a particular piece of media content has been seen by a member of a target audience.

Weekly impressions were calculated in the following manner: for each unique TV ad, each unique TV ad’s average rating was first multiplied by the estimated number of children in the Colombian population. The child population estimate used by Kantar IBOPE was based on projections from the Colombian Administrative Department of Statistics (DANE), which indicated a projected 2 470 700 Colombian children between 4 and 11 years old in 2017. We combined the population data with the ratings data to estimate exposure based on the number of children reached.

The product of each unique TV ad’s average rating and the child population estimate was then multiplied by the TV ad’s number of instances, to incorporate the frequency of exposure in our calculation. The product of this multiplication yielded the total of impressions across all instances of the unique ad. This result was then divided by 52 to determine the average weekly impressions for that unique TV ad. This measure of average weekly impressions not only incorporates the average number of children who were reached during a week but also how frequently that TV ad was shown in a typical week. Therefore, average weekly impressions provide an estimate that includes the two components of exposure: reach and frequency. Following this procedure, we obtained a database with information containing the level of exposure of children aged 4–11 years to food and beverage TV ads in Colombia in 2017.

Finally, we selected the 20 % of ads with the highest average weekly impressions across the overall child audience for the content analysis (591 TV ads). A total of 779 digital recordings were obtained from Kantar IBOPE Media because all versions of the same TV ad were also acquired. However, only the extended versions of those TV ads that had more than one version were included in the sample used for the content analysis.

### Content analysis sample

A total of 576 TV ads were initially included in the sample of TV ads, after filtering out those corresponding to alcoholic beverages and cooking ingredients. From this total, 547 corresponded to TV ads of products available for purchase in Colombia. TV ads of products available for purchase in Colombia promoted a total of 1091 food and beverages products. A total of 16 TV ads, corresponding to 78 products, were excluded either because there was no specific nutritional information available for those products or because there was no specific product identified in the TV ad and therefore no nutritional information could be assigned. The analyses were performed using a final sample of 531 TV ads promoting a total of 1013 food products. Prior to analysis, nutritional information was assigned to each of these food products (see more details about this process in the ‘Linking of advertising content to nutritional data’ section below). Products were then categorised as ‘high-in’ or ‘not high-in’ based on the Pan American Health Organization (PAHO) nutritional criteria described below.

### Marketing codes

Ads were analysed for the use of marketing appeals that represented either emotional or rational persuasive strategies. To do this, existing marketing strategies codebooks^(20,26)^, were first adapted to identify different marketing appeals, for example health claims, quality claims, and use of cartoon characters or animated animals. The categorisation of these appeals as reflecting emotional or rational strategies was informed by prior content analyses that defined different types of appeals using these categoriess^(33,34)^. We use the term “appeal type” to denote individual claims (e.g. quality, nutrition) and tactics (e.g. characters, sports) that comprise emotional and rational persuasive strategies. Table 1 includes the names and definitions of each appeal type within each of the rational and emotional appeal strategies.

**Table 1:** Definition of rational and emotional appeals

Emotional appeals			
*Game*	Kid playing or mention of playing	*Sport game*	Kid on screen performing any activity related with a sport, or any mention of it
*Character*	Kid wearing a costume or acting as costume character	*Licensed character*	Presence of a licensed character from TV, movies, comics or books
*Cartoon*	Cartoon character or any presence of animations	*Celebrity*	Presence of a music, TV or movies celebrity
*Animated animal*	Presence of an animated animal	*Festivals*	Representation of festival or special holiday
*Real animal*	Presence of a real animal	*Sports events*	Representation or mention of a sports event or championship
*Athlete, team, team mascot*	Presence of a professional athlete, elements from a sports team, team logo, mascot or mention of a professional sports team	*Winning*	Mentions of power, speed, energy and winning
*Sportsman or woman represented*	Actor representing a sportsman or sportswoman	*Romance*	Mentions or situations related to sex, romance or physical attractiveness
*Vocabulary*	Use of certain vocabulary or slang popular among youth	*Deregulated consumption*	Incitement to deregulated consumption
*Senses*	Mentions of taste, texture or smell	*Social status*	Mentions of popularity, social status or peer acceptance

*Positive emotions*	Mentions of how consumption can positively alter emotions and mental state	*Negative emotions*	Mentions of how consumption can improve negative emotions or mental states
Rational appeals			
*Quality*	Mentions of awards or certifications given to the product or brand	*Cost*	Allusion to low cost of product
*Brand comparison*	Any mention or information about better quality of product compared with others	*Health and nutrition*	Mentions about the positive nutritional and health qualities of product
*Freshness*	Allusions to the fresh and natural origin of the product	*Body health*	Mentions of how consumption contributes to better health
*Expert approval*	Mentions to how experts recommend or approve consumption	*Ingredients*	Mentions or claims about the presence of healthy ingredients
*Absence of critical nutrients*	Mentions to the lower levels or absence of critical ingredients, such as fat or added sugar		

The codebook was used to identify the presence or absence of different types of rational and emotional appeal strategies in each ad. When a TV ad included in its content a particular type of appeal according to the definitions of each appeal type in the codebook, a score of ‘1’ was typed into that code for that TV ad. This procedure was used to determine the percentage of ads featuring each specific appeal type coded and to determine the percentage of ads featuring any emotional appeal and any rational appeal type.

This procedure was also used to create scores representing the variety of emotional and rational appeal types in each TV ad to determine which of the two appeal strategies were the most frequent among the TV ads in the sample. Variety of appeal type is defined as the extent to which different types of appeals were employed in a TV ad corresponding to each of the two analysed persuasive strategies.

First, the total number of different emotional appeals and the total number of different rational appeal types were calculated for each ad. Then, an ordinal scale for each appeal strategy was created as follows. For each appeal strategy (emotional or rational), when an ad had a total of zero appeal types, it was recorded as 0 for that persuasive strategy. This same procedure was followed for those having one, two or three different appeal types (recoded as 1, 2 or 3, respectively). When a TV ad had four or more different appeal types, it received a score of 4. Therefore, variety of appeal type was measured as an ordinal variable ranging from 0 to 4.

### Coding procedure

A total of eighty food and beverages TV ads from the total sample were randomly selected for training purposes. This number is considered adequate to sufficiently train the coders, as well as to informally evaluate reliability during coder training^(35)^. Two coders were trained over 6 weeks. TV ads used for training were not included as part of the sample for the content analysis reported below (see Table 2). During the training sessions, coders watched and coded the TV ads together. Informal assessments of reliability were carried out, followed by modifications to the codebook were deemed necessary.

**Table 2:** Percentage of products promoted in ads based on the variety of appeal types featured in the ad

Variety of appeals types	Products promoted based on number of different appeals		
	High-in products (*n* 786)	Not high-in products (*n* 227)	Total products (*n* 1013)
Emotional appeal types			
Zero	10·7	25·1	13·9
One	27·9	24·2	27·0
Two	30·0	33·9	30·9
Three	17·3	10·1	15·7
Four or more	14·1	6·6	12·4
Rational appeal types			
Zero	41·5	45·4	42·3
One	19·2	24·2	20·3
Two	21·6	15·0	20·1
Three	11·8	7·9	11·0
Four or more	5·9	7·5	6·2

An initial pilot test for inter-coder reliability assessment was conducted immediately after the main training sessions, in which both coders independently coded a random sample of over 10 % of the ads in the sample (*n* 59), following recommendations for formal and informal reliability assessments in content analysis studies^(34)^. This initial reliability assessment not only revealed relatively acceptable percentage agreement across codes (between 73 % and 100 %) but also allowed us to identify codes that required additional refinement of the coding instrument and coder training due to their rare presence across the ads. After codebook modifications and additional training, a second inter-coder reliability assessment was conducted with a different random sample (*n* 59). Percentage agreement improved across codes (91·37–100 %). The use of other reliability coefficients in addition to percentage of agreement is recommended as percentage of agreement can inflate reliability^(36)^. Thus, Cohen’s Kappa was calculated using the STATA software package to formally assess inter-coder reliability across codes. This inter-coder reliability coefficient has the advantage of providing the level of agreement corrected by chance for nominal scale variables. The combination of scores for percentage of agreement and Cohen’s Kappa coefficients (κ = 0·70–1) indicated acceptable reliability for all codes^(35,36)^. Coders split the remaining ads and coded them independently.

### Linking of advertising content to nutritional data

In addition to marketing appeal codes, we examined each TV advertising for the presence of any additional food or beverage products that were not listed in the products promoted in the TV advertising data provided by Kantar IBOPE. A TV ad was determined as promoting an additional non-listed product if that product was noticeable to the eye and was either named or able to be recognised on the screen. All products promoted by a TV ad, whether listed or non-listed, were assigned a nutritional profile based on their nutritional content.

Products’ nutritional information had been collected in 2018 for another study, in which nutritionists recorded nutritional data from nutrition fact panels and other labels on food and beverage packages available for purchase in major grocery stores in Colombia. After matching advertised products with their nutritional data, we applied the PAHO nutrient profile model^(1)^ to each product with the goal of establishing its nutritional quality.

Each product was classified according to the PAHO model as (1) unprocessed or minimally processed; (2) culinary ingredients; (3) processed or (4) ultra-processed products. Products classified as processed or ultra-processed were further classified as a ‘high in’ product if they exceeded any of the following PAHO thresholds: (1) ≥ 10 % of total energy from free sugars; (2) ≥ 30 % of total energy from total fat; (3) ≥ 10 % of total energy from saturated fats; (4) ≥ 1 % of total energy from trans-fat; (5) ≥ 1 mg of Na per 1 kcal of product or (6) any presence of a non-caloric sweetener among the ingredients.

### Analysis

Descriptive statistics and a Wilcoxon signed-rank test were used to address the first research question. Descriptive statistics show the extent to which high-in and not-high-in products were advertised using emotional and rational appeals, the frequency of each emotional and rational appeal types, as well as the variety of emotional and rational appeal types used to advertise high-in, non-high-in and all products. The Wilcoxon signed-rank test was performed to determine whether a statistically significant difference existed in the variety of emotional and rational appeal types used across all the TV ads.

The second research question was addressed with a Mann–Whitney U test, which allowed us to examine whether high-in products employed a higher variety of rational and emotional appeal types in the TV ads promoting them, compared with non-high-in products.

Research questions three and four were addressed with paired *t* tests to compare exposure to different types of rational and of emotional appeals between female *v*. male children and between children from low, middle and high SES.

## Results

Descriptive statistics in online Supplementary Table S1 show what were the most frequently used persuasive appeal strategies. This table includes the percentage of ads with at least one high-in product, ads with no high-in products and ads in total that featured each type of marketing appeal. The most prevalent appeal type was the ‘senses’ appeal, used to promote 43·0 %, 45·8 % and 33·5 % of products in total, high-in products and non-high-in products, respectively. Also prevalent was the use of cartoons (27·1 % of all products, 28·8 % of high-in products) and positive emotion appeals (29·5 % of all products, 31·8 % of high-in products). Not shown in this table, 52·41 % of products were promoted in ads that featured both an emotional appeal and a rational appeal. Within high-in products, 54·6 % were featured in ads with both types of appeals. Only 8·68 % of products in total and 6·7 % of high-in products were promoted in ads that did not use an appeal we included in our codebook.

The same table also shows the extent to which high-in, non-high-in and total products were promoted with emotional or rational marketing appeal strategies. Specifically, most high-in (89·3 %), non-high-in (74·9 %) and total of products (86·1 %) were promoted in ads that featured at least one emotional appeal type. To a lesser extent, 57·7 % of products in total, 58·5 % of high-in products and 54·6 % of non-high-in products were promoted in ads with at least one of the rational appeals coded.

Descriptive statistics on Table 2 illustrate the frequency in the variety of each persuasive strategy. This table shows the percentage of products in total, high-in products and non-high-in products that were promoted with 0, 1, 2, 3 or 4 or more different types of emotional or rational appeals and reveal that TV ads tended to include a greater variety of emotional appeals than rational appeals.

The Wilcoxon signed-rank test complements the results of the descriptive analyses on Table 2. This test looked at whether the variety of emotional appeal types and the variety of rational appeal types used in the TV ads differed. Results of the Wilcoxon signed-rank test showed that across all TV ads, the variety of emotional appeal types used (*Mdn =* 2) was significantly higher than the variety of rational appeal types used (*Mdn =* 1) (*t* = 22 692·55, *z* = –7·344, *P* < 0·001). In sum, the descriptive and Wilcoxon signed-rank tests showed that the emotional persuasive appeal strategies tended to be used more frequently in the TV ads of food and beverages to which Colombian children were more exposed in 2017.

Subsequent analyses sought to determine whether the TV ads promoting high-in and non-high products differed in the variety of emotional and rational appeal types used. Descriptive statistics in Table 2 show, whereas 31·4 % of high-in products were promoted with 3 or more different emotional appeals, just 16·7 % of non-high-in products. Furthermore, only 10·7 % of high-in products were promoted without any emotional appeals, compared with 25·1 % of non-high-in products. Mann–Whitney U tests, with variety of emotional and rational appeal types defined as ordinal variables, were employed to determine if differences were statistically significant.

Results revealed that there was not a significant difference between high-in and non-high-in products in terms of the variety of rational appeal types used to promote them in the TV ads, independent samples Mann–Whitney U test (*P* = 0·118), (U(N_high-in_ = 786, N_not high-in_ = 227) = 94 988·50, *z* = 1·56, *P* = 0·118). However, for variety of emotional appeals, the analyses showed that more high-in foods and beverages were featured in TV ads that included a higher variety of emotional appeals, compared with non-high-in products (U(*N*_high-in_ = 786, *N*_not high-in_ = 227) = 108 050·00, *z* = 4·99, *P* < 0·001). Thus, significantly more emotional appeals were used to promote high-in than non-high-in products.

### Children’s exposure to different marketing strategies

Given that 77·6 % of the products promoted across ads qualified as ‘high-in’ and that significantly more emotional appeals were used to promote high-in than non-high-in products, the next analyses used TV ads as the unit of analysis (*n* 531) to examine children’s ad exposure based on the extent to which ads featured emotional and rational appeals.

Average weekly impressions equalled 281 802·39, which represents the average number of times that the top 20 % most watched commercials were viewed by Colombian children in 2017. As mentioned previously, the ratings data set also provided audience rating points by gender and for children in low, middle and high SES households. Colombia uses information about external and internal characteristics of a dwelling and its surroundings to determine a stratum for each dwelling. Households can be in one of six possible strata. In the data acquired from Kantar IBOPE Media, low referred to households in strata 1, 2 or 3; middle to households in stratum 4 and high to those in strata 5 and 6.

Since the data set also provided rating points for each TV ad instance for male and female children and for children based on SES, average rating points per TV ad and average weekly impressions were calculated for male and female children, and for children in low, mid and high SES. Following data from DANE, we estimated the population of female children between ages 4 and 11 to be 1 260 057 and male children in the same age range to be 1 210 643. An estimated 1 235 350 children in this age range live in low SES households; 938 866 in middle SES and 444 726 in high SES households. Average weekly impressions were calculated using the same procedure used for overall children. This procedure was explained in the description of the sample. Average weekly impressions were recorded for all products advertised in all the content analysed TV ads.

Paired *t* tests were next used to examine differences in exposure to ads with emotional and rational appeals between female and male children and between children in low, middle and high SES as a means of inferring disproportionate exposure to high-in product promotion. Tables 3 and 4 show the results for these analyses. Male children’s weekly average impressions were first compared with female children’s weekly average impressions for ads with one, with two, with three and with four or more emotional appeal types (i.e. variety of emotional appeal type). The same procedure was done for ads with differing numbers of rational appeal types (i.e. variety of rational appeal type). Weekly impressions for female children were significantly higher than weekly impressions for male children for ads with no emotional appeals, ads with one type of emotional appeal, ads with two types of emotional appeals and ads with three types of emotional appeals. Impressions for male and female children did not differ for ads with four or more emotional appeal types. These findings suggest females had greater exposure to ads generally and to ads with emotional appeals, compared with males. A similar pattern was seen in the analyses with rational appeal types (see Table 3).

**Table 3:** Difference between male and female children exposure to marketing strategy at all appeal level

Ads based on appeal	Different types of appeal (*n of ads*)	*M* Male (sd) – *M* Female (sd) = *M* difference (sd)
Number of emotional appeals	Zero (*n* 77)	118 113·76 (106 440·82) – 127 729·94 (112 662·89) = –9616·18 (35 344·10)*
	One (*n* 145)	141 100·93 (149 227·01) – 150 759·22 (155 988·87) = –9658·29(29 160·89)***
	Two (*n* 155)	159 983·43 (142 178·93) – 167 720·79 (140 203·69) = –7737·36 (38 715·68)*
	Three (*n* 90)	135 833·12 (148 941·24) – 152 571 (184 686·61) = –16,738·46 (45 784·37)***
	Four or more (*n* 64)	124 276·86 (125 781·13) – 125 365·81 (133 519·27) = –1088·94 (27 419·98
Number of rational appeals	Zero (*n* 205)	119 757·01 (89 454·25) – 130 610·02 (100 530·27) = –10,853·01 (35 065·64)***
	One (*n* 120)	135 811·98 (137 882·33) – 136 243·13 (131 775·84) = –431·15 (27 430·63)
	Two (*n* 107)	168 410·47 (155 600·19) – 176 731·93 (162 705·63) = –8321·46 (26 605·41)**
	Three (*n* 55)	123 205·72 (97 594·95) – 140 741·68 (103 952·89) = –17,535·96 (43 752·34)**
	Four or more (*n* 44)	201 969·66 (261 290·21) – 219 807·49 (299 913·99) = –17,837·83 (60 414·34)

* *P* < 0·05.

** *P* < 0·01.

*** *P* < 0·001.

**Table 4:** Differences in exposure between children in low, middle and high SES to marketing strategies across levels of appeal

		*t* test (df)		
Appeal type	Appeal level (*n*)	*M*_Low SES_ (sd) – *M*_Middle SES_ (sd)	*M*_Low SES_ (sd)– *M*_High SES_ (sd)	*M*_Middle SES_ (sd) – *M*_High SES_ (sd)
Emotional	Zero (*n* 77)	165 763·49 (147 693·26) – 56 009·77 (52 217·59) = 109 753·71 (100 446·78)	165 763·49 (147 693·26) – 24 661·62 (20 076·50) = 141 101·87 (129 477·92)	56 009·77 (52 217·59) – 24 661·62 (20 076·50) = 31 348·15 (33 419·27)
	One (*n* 145)	141 100·93 (149 227·006) – 150 759·22 (155 988·87) = 126 461·58 (129 511·82)	141 100·93 (149 227·006) – 68 171·91 (74 670·10) = 164 829·05 (169 058·51)	126 461·58 (129 511·82) – 164 829·05 (169 058·51) = 38 367·46 (42 232·96)
	Two (*n* 155)	219 887·38 (189 391·64) – 75 527·30 (66 386·58) = 144 360·07 (129 505·12)	219 887·38 (189 391·64) – 33 574·94 (30 672·60) = 186 312·44 (163 430·80)	75 527·30 (66 386·58) – 33 574·94 (30 672·60) = 41 952·36 (37 792·71)
	Three (*n* 90)	191 811·63 (230 898·86) – 66 966·60 (72 287·96) = 124 845·03 (160 719·97)	191 811·63 (230 898·86) – 30 874·44 (32 123·41) = 160 937·18 (200 569·01	66 966·60 (72 287·96) – 30 874·44 (32 123·41) = 36 092·15 (41 500·46)
	Four or more (*n* 64)	159 442·16 (164 443·23) – 61 910·58 (64 890·22) = 97 531·57 (103 123·79)	159 442·16 (164 443·23) – 29 107·93 (32 557·18) = 130 334·23 (135 271·15)	61 910·58 (64 890·22) – 29 107·93 (32 557·18) = 32 802·65 (34 412·42)
Rational	Zero (*n* 205)	167 146·41 (129 806·71) – 57 185·56 (41 790·82) = 110 230·85 (92 710·07)	167 416·41 (129 806·71) – 26 276·21 (20 000·56) = 141 140·20 (112 822·02)	57 185·56 (41 790·82) – 26 276·21 (20 000·56) = 30 909·35 (23 756·43)
	One (*n* 120)	179 897·05 (179 475·84) – 64 731·06 (65 399·55) = 115 165·98 (117 638·94)	179 897·05 (179 475·84) – 28 475·46 (28 438·13) = 151 422·59 (154 281·51)	64 731·06 (65 399·55) – 28 475·46 (28 438·13) = 36 256·60 (38 967·28)
	Two (*n* 107)	231 250·07 (212 862·47) – 80 832·64 (75 934·44) = 150 414·43 (142 193·89)	231 250·07 (212 862·47) – 35 260·98 (34 069·37) = 195 989·09 (182 371·93)	80 832·64 (75 934·44) – 35 260·98 (34 069·37) = 45 571·66 (43 466·29)
	Three (*n* 55)	170 105·08 (120 981·13) – 63 698·55 (55 710·02) = 106 406·52 (77 261·86)	170 105·08 (120 981·13) – 30 308·92 (29 097·02) = 139 796·15 (100 375·98)	63 698·55 (55 710·02) – 30 308·92 (29 097·02) = 33 389·63 (29 005)
	Four or more (*n* 44)	284 726·96 (375 417·29) – 96 600·05 (129 180·13) = 188 126·90 (249 319·44)	284 726·96 (375 417·29) – 41 428·15 (57 384·61) = 243 298·80 (319 295·07)	96 600·05 (129 180·13) – 41 428·15 (57 384·61) = 55 171·90 (72 335·85)

All *t* tests are significant at the *P* <0·001 level.

The average weekly exposure to emotional and rational appeal types at all levels of both appeal strategies increased as SES decreased is shown in Table 4. Children in low SES households had significantly higher levels of exposure to ads generally, compared with children in middle or high SES households. Children in middle SES households had significantly more exposure to food and beverage ads, compared with children in high SES households. Therefore, exposure to a higher variety of emotional and rational appeal types was present among children in lower SES.

## Discussion

These results expand current knowledge on the degree to which children are exposed to high-caloric and energy dense food product advertising and which strategies are most frequently used to persuade these audiences in the Latin American region.

Our analyses showed that emotional appeals are used more frequently than rational appeals on children’s most watched TV food ads. Furthermore, we found that emotional appeals were more often used to advertise ‘high-in’ products than ‘non-high in’ food and beverage products. Results showed that the distribution of type of appeal among the 20 % TV ads of food and beverages most viewed by children significantly differed. On an average week, the most viewed TV ads tended to have more emotional than rational appeals. The finding is not surprising, as these types of marketing tactics have been shown to be more effective in influencing audiences, particularly children^(22)^. Due to their developmental stage, children are driven by reward and gratification and have difficulty with understanding and assessing risk, particularly if it relates to future consequences, which makes them vulnerable to marketing that suggests fun or enjoyment^(37)^. Furthermore, children’s cognitive development is influenced heavily by observational learning, so this learning style coupled with the importance of friendship and social acceptance combines to make children vulnerable to messages that also use child actors or child-appealing characters that model consumption behaviours or suggest social norms of consumption^(12,24)^.

Even if motivated and able to carefully process the information in the food ads, children would not have been presented with ample rational appeals about the nutritional quality of the products to be able to ‘balance out’ the potential effect of the emotional appeals used to promote the products. Whereas most of the TV food ads we observed included at least one or two emotional appeals, many of the ads observed have between zero or no rational appeals. Given research showing that advertising literacy and executive function might moderate the potential effects of TV ads, future research is needed to examine whether improving children’s literacy for understanding advertising would be effective for ads that emphasise emotional rather than rational appeals.

The higher usage of emotional appeals in high-in food advertising, specifically, also poses important challenges when considering how they might combine with rational appeals in the ads. Based on our analyses, the most used rational appeals referred to the health and nutritional advantages of the product, its freshness and its ingredients. Mentions of rational appeals did not differ between those products exceeding and those not exceeding recommended levels of critical nutrients. Thus, independent of the nutritional qualities of a food product, TV ads tended to include the same number of claims about the nutritional and health advantages of the product. The most prevalent emotional appeals used cartoons or included claims about senses or positive emotions related to the consumption of the product. Given the co-occurrence of rational and emotional appeals in high-in products, research is needed to determine how children might be making associations between the positive emotions, experiences and sensations of consumption with nutritional advantages for products that may pose risks to their health, overweight and obesity levels.

More troubling, disproportionate exposure to TV ads of ‘high-in’ products that use more emotional appeals was also observed when degree of exposure between genders and children from different SES were compared. Results show that in general, female children and lower-SES children are exposed to more food and beverage products advertising. Given obesity in childhood predicts obesity in adulthood, this disproportionate marketing exposure has implications for adult rates of obesity in Colombia, which are higher for women and for adults from lower SES^(31)^. Furthermore, to the extent that girls are likely to have the same eating patterns in childhood as in adulthood and possibly pass these habits to their own children^(38)^, disproportionate marketing exposure in youth might have implications for the association between maternal consumption of high-in foods and increased risk of overweight or obesity in offspring. Future studies are therefore needed to evaluate and better understand the potential impact of higher exposure on females from an early age, and the consequences of this overexposure in adulthood.

The finding with respect to children in lower socio-economic strata is concerning, given previous studies reporting that children from low SES or low-income families are more likely to eat snacks in front of the TV than those from highly affluent families^(39–41)^. Moreover, Coon *et al*. found that children from low-income families are more likely to have the TV on, which is especially shown in single-parent families^(42)^. Yet, in Colombia, excessive screen time affects seven out of ten school children in urban areas, compared with five out of ten in rural areas^(43)^. Therefore, this finding of disproportionate exposure for lower-SES children should be interpreted with the understanding that higher-SES children may have greater access to digital media and might be similarly exposed to unhealthy food marketing across the media they use. Regardless of whether TV is the most important source or merely one of several sources of children’s unhealthy food marketing exposure, it is critical to address unhealthy food marketing as a risk factor for malnutrition, given the predominant use of emotional appeals and problematic use of rational nutrition-related appeals in ultra-processed and other high-in products.

This study does not test the effects on children’s purchasing requests, consumption patterns or family buying patterns. However, extant research shows how advertising influences these types of behaviours, and how children’s eating preferences are shaped by the content of the TV ads to which they are exposed. Although limited in the implications for effects of the content, this study shows relevant patterns in exposure to marketing strategies that have been proved to have a strong effect on children and their health. Another limitation of this study is the focus on cataloguing the nutritional quality of packaged items that appeared in the commercials. Some products, namely unpackaged foods such as pizza, hamburgers and hot dogs, would qualify as fast food but were left out of the analysis due to challenges with identifying product ingredients and therefore nutritional quality. It would be important for future studies to include these types of products in analyses. Thus, it is possible our findings are an underrepresentation of the nutritional quality of foods in our sample of ads.

Despite these limitations, findings of this study underscore the challenge unhealthy food advertising on TV poses for Colombian public health, due to children’s vulnerability to emotion-based marketing strategies and the consequences that high caloric energy-dense food and beverage products have for obesity-related risks and outcomes. Childhood overweight and obesity have been linked to exposure to TV ads^(44)^. A prior study conducted in Colombia found that 89·3 % of the products advertised on TV were for high-in foods and more than 80 % of children from 4 to 11 years of age of all socio-economic levels were exposed to these advertising products^(29)^. The present study further reveals the predominance of emotional appeals in this advertising and the disproportionate exposure of unhealthy food ads with these appeals for female children and children of lower SES. It is well known that food and beverage advertising is a main factor influencing food choices and eating patterns in children, and the exposure patterns make it clear that industry is directing its messages to this vulnerable populations^(45)^. Ample research indicates that increased consumption of high-in foods and beverages is associated with increased intake of energy, free sugars and decreased fibre, increasing the potential risk of obesity in children and adolescents^(46)^ and faster progression of BMI, fat mass index, weight and waist circumference in adolescence and early adulthood^(47)^. Thus, children and adolescents with overweight and obesity are at increased risk of developing non-communicable diseases such as type 2 diabetes, hypertension, CVD, liver disease and other diseases later in life^(46)^.

The present study strengthens our knowledge of the most common food marketing strategies aimed at children in Colombia and demonstrates the importance of regulating these strategies to prevent unhealthy eating habits among children and reduce child malnutrition. It is important to note that, whereas child-oriented marketing government regulations are few around the world^(48)^, numerous studies show the need to implement measures to regulate advertising on all platforms and on each ultra-processed food and beverages^(45)^. So far, Colombian regulations have focused on limiting misleading advertising. This regulation, however, is insufficient because it does not ensure the quality of information on ultra-processed food products^(23,37)^. Yet, given the risk of harm to children and continued risk as they develop into adults, it should be a priority for governments to implement policies that reduce children’s exposure to unhealthy food marketing as one measure in the fight against malnutrition and non-communicable diseases.

## Supplementary material

For supplementary material accompanying this paper visit https://doi.org/10.1017/S1368980023001702. click here to view supplementary material
