# Supplementary material for: Food for thought or food for emotions? An analysis of marketing strategies in television food advertising seen by children in Colombia
Source: Public Health Nutr. 2023 Aug 10;26(11):2243–55. doi: 10.1017/S1368980023001702 (PMC10641607; doi:10.1017/S1368980023001702)
Supplement: Supplementary file 1 [file S1368980023001702sup001.docx]

| Supplementary Table S1. Percentage of products promoted with at least one of each coded emotional or rational appeal | | | |
| --- | --- | --- | --- |
| Marketing appeal | Products promoted with at least one type of appeal | | |
|  | High-in products  (*n* = 786)  % | Not high-in products  (*n* = 227)  % | Total products  (*n* = 1013)  % |
| Any emotional appeal | 89.3 | 74.9 | 86.1 |
| Game | 6.4 | 9.7 | 7.1 |
| Character | 1.7 | 0.0 | 1.3 |
| Cartoon | 28.8 | 21.6 | 27.1 |
| Animated animal | 10.2 | 15.0 | 11.3 |
| Real animal | 5.2 | 4.8 | 5.1 |
| Athlete, team, team mascot | 6.0 | 4.0 | 5.5 |
| Sportsman or woman represented | 4.1 | 4.4 | 4.1 |
| Vocabulary | 18.4 | 2.2 | 14.8 |
| Senses | 45.8 | 33.5 | 43.0 |
| Positive emotions | 31.8 | 21.6 | 29.5 |
| Sport game | 3.3 | 0.9 | 2.8 |
| Licensed character | 1.8 | 0.0 | 1.4 |
| Celebrity | 10.6 | 10.1 | 10.5 |
| Festivals | 3.3 | 2.6 | 3.2 |
| Sports events | 1.0 | 0.4 | .9 |
| Winning | 5.0 | 0.9 | 4.0 |
| Romance | 5.3 | 1.8 | 4.5 |
| Deregulated consumption | 5.1 | 1.3 | 4.2 |
| Social status | 4.8 | 10.6 | 6.1 |
| Negative emotions | 6.7 | 1.3 | 5.5 |
| Any rational appeal | 58.5 | 54.6 | 57.7 |
| Quality | 11.6 | 20.7 | 13.6 |
| Brand comparison | 8.3 | 15.9 | 10.0 |
| Freshness | 23.5 | 15.4 | 21.7 |
| Expert approval | 2.2 | 3.5 | 2.5 |
| Absence of critical nutrients | 6.5 | 8.8 | 7.0 |
| Cost | 8.8 | 4.0 | 7.7 |
| Health and nutrition | 23.0 | 22.0 | 22.8 |
| Body health | 1.0 | 0.9 | 1.0 |
| Ingredients | 37.5 | 19.8 | 33.6 |
